# Efficacy of melatonin in term neonatal models of perinatal hypoxia‐ischaemia

**DOI:** 10.1002/acn3.51559

**Published:** 2022-04-12

**Authors:** Raymand Pang, Hyun Jee Han, Christopher Meehan, Xavier Golay, Suzanne L. Miller, Nicola J. Robertson

**Affiliations:** ^1^ Institute for Women's Health University College London London UK; ^2^ Institute of Neurology, Queen's Square University College London London UK; ^3^ The Ritchie Centre, Translational Research Facility Hudson Institute of Medical Research Clayton Australia; ^4^ Department of Obstetrics and Gynaecology Monash University Clayton Australia; ^5^ Centre for Clinical Brain Sciences University of Edinburgh Edinburgh UK

## Abstract

**Objective:**

Neonatal encephalopathy (NE) is an important cause of mortality and disability worldwide. Therapeutic hypothermia (HT) is an effective therapy, however not all babies benefit. Novel agents are urgently needed to improve outcomes. Melatonin in preclinical studies has promising neuroprotective properties. This meta‐analysis assessed the efficacy of melatonin in term animal models of NE on cerebral infarct size, neurobehavioural tests and cell death.

**Methods:**

A literature search was carried out using Embase, MEDLINE and Web of Science (31 May 2021). We identified 14 studies and performed a meta‐analysis with a random effects model using standardised mean difference (SMD) as the effect size. The risk of bias was assessed using the Systematic Review Centre for Laboratory animal Experimentation tool and publication bias was assessed with funnel plots, and adjusted using trim and fill analysis. Subgroup and meta‐regression analyses were performed to assess the effects of study design variables.

**Results:**

We observed significant reduction in brain infarct size (SMD −2.05, 95% CI [−2.93, −1.16]), improved neurobehavioural outcomes (SMD −0.86, 95% CI [−1.23, −0.53]) and reduction in cell death (SMD −0.60, 95% CI [−1.06, −0.14]) favouring treatment with melatonin. Neuroprotection was evident as a single therapy and combined with HT. Subgroup analysis showed greater efficacy with melatonin given before or immediately after injury and with ethanol excipients. The overall effect size remained robust even after adjustment for publication bias.

**Interpretation:**

These studies demonstrate a significant neuroprotective efficacy of melatonin in term neonatal models of hypoxia‐ischaemia, and suggest melatonin is a strong candidate for translation to clinical trials in babies with moderate–severe NE.

## Introduction

Neonatal encephalopathy (NE) is a leading cause of mortality and morbidity in newborns across the world. The regional incidence varies from 2.4 to 2.6 per 1,000 live births in England^1^ to estimates of 14.9 per 1,000 live births in Sub‐Saharan Africa.^2^ This highlights the disproportionately higher burden of disease in low resource settings.^2^

Over several decades, scientific attention has focussed on the development of novel neuroprotective therapies to improve outcomes for infants with NE. Preclinical studies have provided key information advancing our understanding of the evolution of brain injury following hypoxia‐ischaemia (HI)^3^ and have supported the translation of therapeutic hypothermia (HT) into large randomised clinical trials (RCTs) for infants with moderate to severe NE.^4^ While HT is now an integral aspect of neonatal neurocritical care in high resource settings, children still suffer long‐term complications. The rate of cerebral palsy remains static at 14–19% and no significant improvement in IQ or disability was observed at 6–7‐year follow‐up in children who received HT.^5^ The current HT protocol targeting 33.5°C for 72 h is optimal, as confirmed by the studies of deeper and longer cooling.^6^, ^7^ In low‐ and middle‐income countries (LMICs), a recent large RCT suggests no benefit of HT in infants with NE.^8^ Taken together, there is now an urgent need to translate the most promising neuroprotective therapies from preclinical studies to early phase RCTs.

There is compelling preclinical evidence of the safety and efficacy of melatonin as a neuroprotective therapy.^9^, ^10^ Melatonin is an indolamine hormone and a potent free radical scavenger, which removes toxic reactive oxygen species generated following HI.^11^, ^12^, ^13^ Melatonin also exhibits anti‐apoptotic^14^, ^15^ and anti‐inflammatory^16^ actions. Clinical trials of melatonin have so far been limited to small, underpowered studies, lacking consistent neurodevelopmental outcomes.^17^ While preclinical studies provide vital safety and efficacy data to support clinical trials, promising neuroprotective agents in animals have not always translated into a positive biological effect in RCTs.^18^ Several groups have supported the use of preclinical systematic reviews and meta‐analyses to improve the transparency and accessibility to animal data.^19^ Conducting systematic reviews not only provides the ability to synthesise the overall effect size, but also allows scrutiny in the validity of the preclinical evidence. Data from meta‐analyses may highlight gaps in knowledge, and thereby reducing unnecessary duplication leading to improved adherence to the 3Rs (Replacement, Refinement, Reduction).

We carried out a systematic review and meta‐analysis to assess the neuroprotective efficacy of melatonin, with and without HT, in term neonatal animal models of NE. The primary outcome measures of neuroprotective efficacy were gross cerebral infarct size, neurobehavioural outcomes and cell death assessed using histology. These outcomes were chosen as they are commonly reported, robust biomarkers of neuroprotection and neuropathology in animal studies. MacLeod et al. previously reported a 42.8% improvement in outcome with melatonin in adult stroke models^20^ however, the efficacy in neonatal models is unknown. It is critical to assess neuroprotective agents for newborn brain injury in animals of equivalent gestation due to the vulnerability of the yet fully developed brain.

## Materials and Methods

This study was reported in accordance with the updated Preferred Reporting Items for Systematic Reviews and Meta‐Analyses (PRISMA 2020). The review was conducted using the standard Systematic Review Centre for Laboratory animal Experimentation (SYRCLE)^21^ template prior to data collection. While this study was not preregistered on the PROSPERO database, we included the full protocol as a supplementary document. No retrospective alterations to the protocol were made.

### Eligibility criteria

The selection criteria for inclusion in the systematic review were; (1) animal models of near‐term gestation (rodents at ≥ postnatal day 7 [P7], sheep at ≥126 gestational days, newborn piglets) (2) subjected to brain injury with both a hypoxic and ischaemic component, (3) received melatonin (4) compared with normothermic or HT controls (5) with ≥1 of the three predefined outcome measures: (i) overall infarct size, (ii) neurobehavioural outcome and (iii) histological assessment of cell death. Fully peer‐reviewed published and in‐press articles were included. Conference abstracts, preterm models and in‐vitro studies were excluded.

### Search criteria

A literature search was carried out on 31 May 2021 using Ovid Embase, MEDLINE and Clarivate Web of Science (Core Collection) databases using the following search terms; Melatonin AND brain injury OR neonatal encephalopathy OR hypoxia OR hypoxia‐ischaemia OR foetal hypoxia OR perinatal asphyxia OR asphyxia neonatorum OR asphyxia. Please see supplementary document for full search terms. We elected not to search the preprint servers. A previously published^22^ search filter of animal studies for Embase was utilised. No other filters or limits were applied to the search strategy to allow maximal retrieval of results.

### Screening and data extraction

Publication titles and abstracts were screened independently by two reviewers (R. P. and H. J. H.) using the Systemic Review Facility (SyRF) platform (RRID:SCR_018907).^23^ Discrepancies were resolved by a third reviewer (C. M.). Studies meeting the eligibility criteria were subjected to full‐text review. Animal characteristics (age, sex, weight, temperature control), brain injury methodology and quantitative outcome data (mean and error values, number of animals) were extracted. Where the error value was given but the error type not stated, SEM was used as a conservative approach to ensure the effect size was not over‐estimated. For neurobehavioural outcomes, all tests performed were collected. Where the same test was repeated over several intervals, the final time point was used. For cell death markers, values were recorded for each available brain region and total cell counts were excluded to avoid repetition. The melatonin dose over the first 24 h (mg/kg), time of first dose, dosing regimens, route of administration and excipients used were extracted. The dose of melatonin was allometrically scaled to human equivalent doses (HED) for a 3‐kg infant using the simple dose by factor method^24^ to allow comparisons between the species. Data between the reviewers (R. P. and H. J. H.) were compared to ensure consistency and discrepancies were resolved through review of full‐text and discussion until consensus reached. Where data was given in graphical form, the first and last authors were approached.

### Assessment of bias

The risk of bias was assessed for each study by two reviewers (R. P., H. J. H.) independently using the SYRCLE Risk of Bias (RoB) tool. Studies were assessed for selection, performance, detection, attrition and reporting bias and categorised as high or low risk. Where authors did not provide sufficient detail in the manuscript to assess the risk adequately, the risk was categorised as unclear. A final consensus was reached between the two reviewers through a consensus‐orientated discussion.

### Statistical analysis

Statistical analysis was performed using JMP (Version 15, SAS, Marlow, Buckinghamshire, UK), Review Manager (Version 5.4, The Cochrane Collaboration, London, UK) and STATA (version 17, StataCorp, College Station, Texas, USA). Hedge's standardised mean difference (SMD) was used to summarise the effect size, which adjusts for small sample size and accounts for the difference in the scale of measurement. For outcomes with multiple data values available, data were combined into a single, ‘nested outcome’ as described by Vesterinen.^25^ All available primary outcome measures for each study were combined using the same method to allow subgroup analysis.

Subgroup analyses were performed to provide evidence to inform future clinical neuroprotection studies. We used stratified subgroup meta‐analysis for categorical variables and meta‐regression analysis for continuous variables. We firstly assessed the efficacy of melatonin as a monotherapy and as an adjunct agent to HT. Subgroup stratification provided an intuitive method to assess the effect size separately, which is important to support future studies relevant to the low or high resource setting, respectively. Subgroup stratification by melatonin dose (scaled to HED), time of first dose, excipient used in the melatonin formulation were also assessed to inform future studies. The influence of preclinical study design factors including sex, animal species, anaesthetic agent and type of HI brain injury were assessed in the same manner. Significance testing of subgroup difference was performed using the Borenstein method implemented on RevMan. Meta‐regression analysis fitted to a random effects (restricted maximum‐likelihood) model was performed to explore the effect of dose and time of melatonin administration. Similarly, the influence of the study quality on the pooled effect estimate was assessed using the same model with the RoB score as the explanatory variable.

The meta‐analyses were performed to obtain a pooled effect size estimate (95% CI) using the random effects model (DerSimmonian and Laird method) and presented as forest plots, ordered by effect size. This model is commonly used in preclinical studies to account for the expected heterogeneity between animal studies.^25^ Heterogeneity was assessed using chi‐squared test with degrees of freedom and *I* ^2^. A higher significance level of *p* < 0.10 was used to compensate for the low power of the test.^26^ Missing data were assessed by subgroup and meta‐regression analysis. Publication bias of the three outcome measures was explored using a funnel plot of SMD versus SE and adjusted using trim and fill analysis. Forest plot using a sample‐based precision approach (SMD vs. 1/√*n*) was also explored given the susceptibility of standard SMD versus SE funnel plots to overestimate publication bias.^27^ To inform sample size estimates for future neuroprotection studies, power calculations were performed on the cerebral infarct size using the observed 25th, 50th and 75th percentile SMD values with the median SD at 80% power and alpha value of 0.05.

## Results

**Figure 1:**
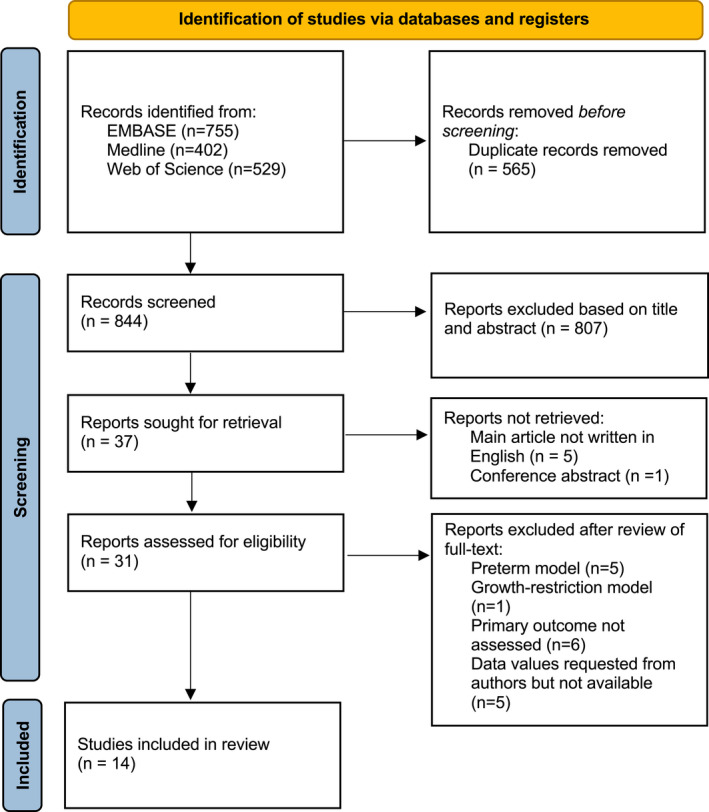
PRISMA flow diagram of the literature search.

A total of 1686 records were identified and after removal of duplicate records (*n* = 565), 844 titles and abstracts were screened for eligibility. The PRISMA flow diagram is shown in Figure 1. Following exclusion of irrelevant records, 31 full‐text articles were retrieved and assessed. Six articles were excluded as they were inappropriate animal models (preterm *n* = 5, intrauterine growth restriction *n* = 1) and six further articles were excluded as the primary outcome measures were not assessed. A further five articles were excluded as primary outcome data were available in graphical form only and authors did not respond to data request. The study characteristics of the remaining 14 studies,^28^, ^29^, ^30^, ^31^, ^32^, ^33^, ^34^, ^35^, ^36^, ^37^, ^38^, ^39^, ^40^, ^41^ which met the inclusion criteria and reported in this meta‐analysis are shown in Table 1. This included six studies with quantitative data made available following data request from corresponding authors.^28^, ^29^, ^30^, ^31^, ^36^, ^41^

**Table 1:** Summary of study characteristics.

Publication	Species, age, sex	HT	Anaesthesia	Brain injury	Dosing regime	Melatonin Dose mg/kg (HED)	Excipient	Melatonin levels (Cmax) mg/L	Duration of experiment (days)	Infarct size	Neurobehavioural tests	Histological assessment of cell death
Carloni (2008)	Rat, P7, NS	No	Isoflurane	Carotid artery ligation, ↓FiO_2_	Intraperitoneal: Pre‐HI, 5 min after HI, 24 h, 48 h	15 (2.6)	5% DMSO	Not reported	60	% ipsilateral injury using toluidine blue staining	Circular maze test	No response to data request
Cetinkaya (2011)	Rat, P7, mixed	No	Isoflurane	Carotid artery ligation ↓FiO_2_	Intraperitoneal: Pre‐HI, immediate after HI, 24 h	40 (7)	10% ethanol	Not reported	3	% infarct volume with TTC	NS	No response to data request
Alonso‐Alconada (2012)	Rat, P7, NS	No	Isoflurane	Carotid artery ligation ↓FiO_2_	Intraperitoneal: 5 mins after HI, 24 h, 48 h	15 (2.6)	5% DMSO	Not reported	7	Mean left:right hemisphere area ratio using cresyl violet staining	NS	qualitative only
Ozyener (2012)	Rat, P7, mixed	No	Isoflurane	Carotid artery ligation ↓FiO_2_	Intraperitoneal: Pre HI, immediate after HI, 24 h	40 (7)	10% ethanol	Not reported	3	% infarct volume with TTC	NS	No response to data request
Yawno (2012)	Lamb, 126 days, mixed	No	Halothane	Umbilical cord occlusion	intravenous: 2 h before HI	0.75 (0.82)	1% ethanol	Not reported	2	NS	NS	CC3
Robertson (2013)	Piglet, <48 h old, Male	Yes	Isoflurane	Carotid artery ligation ↓FiO_2_	Intravenous Infusion over 6 h: Immediately after HI, 24 h	30 (26.2)	2.5% ethanol	21	2	NS	NS	TUNEL
Revuelta (2017)	Rat, P7, NS	No	Isoflurane	Carotid artery ligation ↓FiO_2_	Intraperitoneal: after HI	15 (2.6)	5% DMSO	Not reported	7	NS	NS	NeuN
Aridas (2018)	Lamb, 139–141 l days, mixed	No	Sodium thiopentone	Umbilical cord occlusion	Intravenous boluses of 5 mg every 2 h, from 30 min to 24.5 h after HI	15 (16.5)	3% ethanol	<1	3	NS	Feeding, suckle, tone, standing	CC3
Berger (2019)	Rat, P7, mixed	No	Isoflurane	Carotid artery ligation ↓FiO_2_	Intraperitoneal: After HI, 6 h, 25 h	20 (3.5)	5% DMSO	Not reported	43	NS	Cylinder test, novel object recognition	H&E
Robertson (2019)	Piglet, <48 h old, male	Yes	Isoflurane	Carotid artery ligation ↓FiO_2_	Intravenous 2 h infusion: 1 h after HI, 26 h	15 (13.1)	Ehanol‐free	16.8	2	NS	NS	TUNEL
Robertson (2020)	Piglet, <48 h old, male	Yes	Isoflurane	Carotid artery ligation ↓FiO_2_	Intravenous 6 h infusion: 1 h after HI, 24 h	18 (15.7)	2.5% ethanol	18.8	2	NS	NS	TUNEL
Aridas (2021)	Lamb, 139–141 days, mixed	Both	Sodium Thiopentone	Umbilical cord occlusion	Intravenous boluses of 5 mg every 2 h, 30 min to 24.5 h after HI	15 (16.5)	5% ethanol	<1	3	NS	Standing, feeding	CC3
Pang (2021)	Piglet, <48 h old, male	Yes	Isoflurane	Carotid artery ligation ↓FiO_2_	Intravenous 2 h infusion: 1 h after HI, 24 h, 48 h	20 (17.5)	Ehanol‐free	27.8	3	NS	NS	TUNEL
Sun (2021)	Mouse, P10, mixed	No	Diethyl ether	Carotid artery ligation ↓FiO_2_	Intravenous boluses of 5 mg every 2 h, 30 min to 24.5 h after HI: Immediately after HI, repeated every 24 h for 28 days	10 (1.2)	3% tween	NS	28	% infarct volume with TTC	Negative geotaxis, cliff avoidance, Forelimb suspension, surface righting, novel object recognition, open field, step‐through, foot fault, cylinder tests	NS

CC3, cleaved‐caspase‐3; DMSO, diethyl sulfoxide; H&E, haematoxylin and eosin; HT, hypothermia; HED, human equivalent dose; NeuN, neuronal nuclei; TTC, triphenyltetrazolium chloride; TUNEL, terminal deoxynucleotidyl transferase dUTP nick end labelling; NS, not stated.

### Study characteristics

Included in this meta‐analysis were seven small animal (P7 rats *n* = 6, P10 mice *n* = 1), and seven large animal (lamb *n* = 3, piglets *n* = 4) studies. Four studies assessed the efficacy of melatonin in combination with HT whereas nine studies were in normothermic animals. One study assessed the efficacy of melatonin in both HT and normothermic animals, so was subdivided into two comparative studies.

Melatonin dosing regimens varied across the studies (Table 1): four studies administered the first dose before HI, five studies immediately after HI, and five studies delayed by 30 min–2 h after HI. Melatonin HED ranged from 0.8 to 26.2 mg/kg. Most studies used ethanol as the excipient (*n* = 7), four studies used 5% dimethyl sulfoxide (DMSO), two studies used an ethanol‐free excipient developed by *Chiesi* and one study used 3% Tween. Pharmacokinetics studies were reported in six studies, with peak melatonin levels of <1 mg/L in two lamb studies, 15–20 mg/L in two piglet studies levels of >20 mg/L in two piglet studies.

Five studies reported an overall cerebral infarct size, five studies reported neurobehavioural outcomes and nine reported histological assessment of cell death. For histology, four studies reported terminal deoxynucleotidyl transferase dUTP nick end labelling (TUNEL), three studies reported cleaved‐caspase 3, one study reported a histology injury score based on neuronal loss from haematoxylin‐eosin staining and one study reported NeuN+ cell counts. Histology data from three studies were not included in this review as the authors did not respond to data request.

### Overall effect size

**Figure 2:**
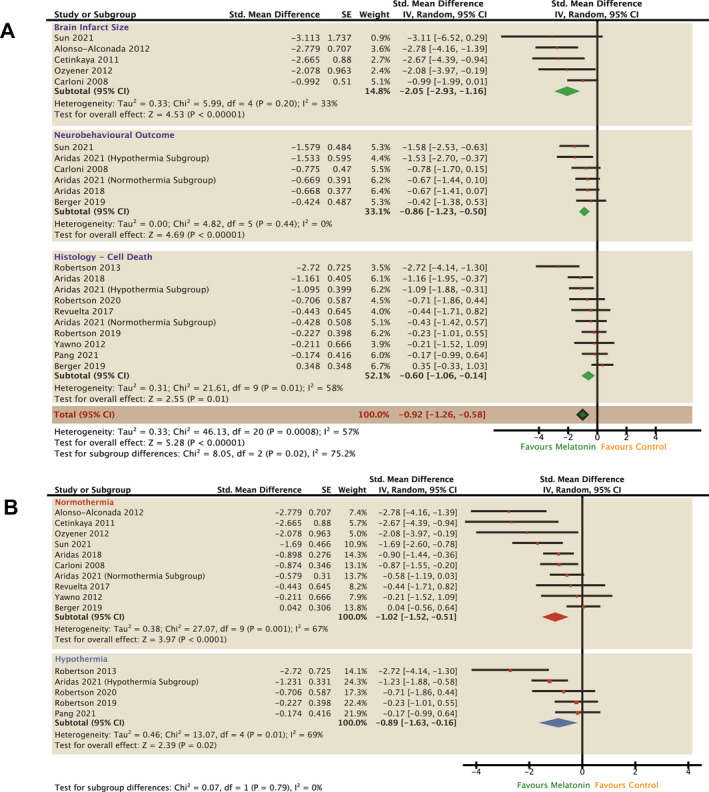
Effect of melatonin on gross cerebral infarct size, neurobehavioural outcome and cell death (A). Further stratified subgroup analysis to assess the efficacy of melatonin as a single therapy or as an adjunct agent with HT on combined neurological outcome (B). Forest plots of SMD with 95% CI ordered by effect size. Pooled estimate of effect size calculated using random effects model. SMD, standardised mean difference; HT, hypothermia.

Meta‐analysis was performed on brain infarct size, neurobehavioural outcomes and histological assessment of cell death (Fig. 2A). In melatonin treated animals, we observed significant reduction in brain infarct size (pooled SMD estimate −2.05, 95% CI [−2.93 to −1.16], *p* < 0.001, *n* = 110 animals), improved neurobehavioural outcomes (SMD −0.86, 95% CI [−1.23 to −0.50], *p* < 0.001, *n* = 141 animals) and reduction in cell death on histology (SMD −0.60, 95% CI [−1.06 to −0.14], *p* = 0.01, *n* = 207 animals) compared to untreated controls. The interstudy heterogeneity was low for brain infarct size (*χ* ^2^ = 5.99, *p* = 0.2, *I* ^2^ = 33%) and neurobehavioural outcomes (*χ* ^2^ = 4.82, *p* = 0.44, *I* ^2^ = 0%) and moderate for histology (*χ* ^2^ = 21.61, *p* = 0.01, *I* ^2^ = 58%). The pooled estimate of effect size remained significant when all three outcomes were combined (SMD −0.92, 95% CI [−1.26 to −0.58], *p* = 0.02) with moderate heterogeneity (*χ* ^2^ = 46.13, df = 20, *p* < 0.001, *I* ^2^ = 57%).

Subgroup analysis was performed to assess the efficacy of melatonin as a single agent in normothermic animals and as an adjunct to HT (Fig. 2B). As a single agent, melatonin was associated with a significant improvement in combined outcomes (SMD −1.02, 95% CI [−1.52 to −0.51], *p* < 0.001, *I* ^2^ = 67%). Melatonin in combination with HT was also associated with a significant improvement in outcomes (SMD −0.89, 95% [−1.63 to −0.16], *p* = 0.02, *I* ^2^ = 69%) compared to HT alone. The test for subgroup differences was not significant (*p* = 0.79).

### Melatonin regimens

**Figure 3:**
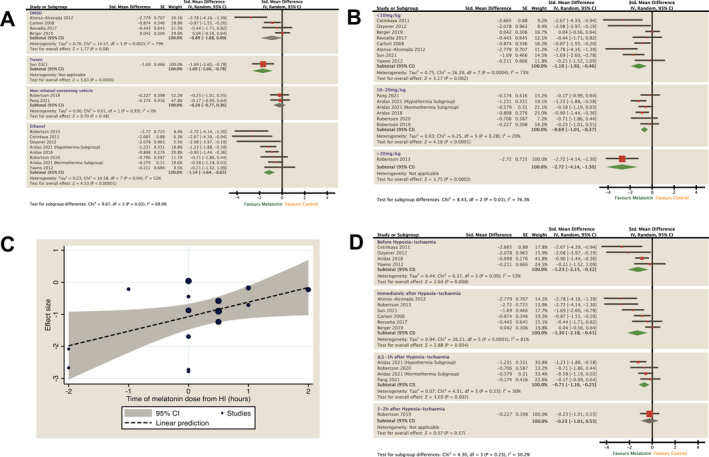
Subgroup analysis to assess the effect of excipients (A), time of melatonin administration (B and C) and allometrically scaled HED of melatonin (D) on combined neurological outcome. Forest plots of SMD with 95% CI ordered by effect size (A and D) or dose (B). Association between time to dose of melatonin from HI and effect size shown as bubble plot (C) with trend line and 95% CI after meta‐regression analysis. HED, human equivalent doses; SMD, standardised mean difference; HI, hypoxia‐ischaemia.

Melatonin administration regimens (Table 1) were compared using subgroup analysis (Fig. 3). Melatonin formulations containing ethanol were most protective (SMD −1.14, 95% CI [−1.64 to −0.65], *p* < 0.001, *I* ^2^ = 52%) whereas we observed no significant efficacy in studies using the non‐ethanol excipient (SMD −0.2, 95% CI [−0.77 to 0.36], *p* = 0.93, *I* ^2^ = 0%) or DMSO (−0.89, 95% CI [−1.88 to 0.09], *p* = 0.002, *I* ^2^ = 79%) (Fig. 3A). Neuroprotection was also observed in melatonin dissolved in Tween (SMD −1.69, 95% CI [−2.60 to −0.78], *p* < 0.001) from one study.

Early melatonin administration was associated with a greater degree of improvement in pooled outcomes (Fig. 3D). We observed the greatest efficacy in animals who received melatonin before HI (SMD −1.23, 95% CI −2.15 to −0.32, *I* ^2^ = 53%) and immediately (5–30 min) after HI (SMD – 1.3, 95% CI −2.18 to −0.41, *I* ^2^ = 81%). Efficacy reduced when melatonin was given after 1 h (−0.71, 95% CI −1.16 to −0.25, *I* ^2^ = 30%) and no significant effect was observed in one study where melatonin was given after a delay of 2 h (SMD −0.23 [95% CI −1.01 to 0.55]). Meta‐regression analysis showed a significant positive correlation between time of melatonin administration from HI and reduction in pooled effect size (co‐efficient 0.45, 95% CI [0.15–0.89], *p* = 0.043, *r* ^2^ = 0.252) (Fig. 3C). We observed improved outcomes in all melatonin doses and meta‐regression analysis showed no significant correlation between melatonin dose and effect size (*r* = −0.01, 95% CI [−0.07 to 0.05], *r* ^2^ = 0.00) (Fig. 3B).

### Study characteristics

**Figure 4:**
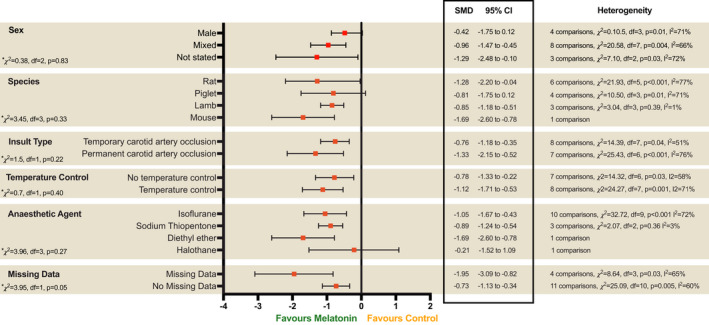
Subgroup analysis to assess the influence of study characteristics. Forest plots of SMD with 95% CI. Test of subgroup differences where *p* > 0.1 represents statistical significance.

Stratified subgroup analysis was performed to assess the influence of study design on outcomes (Fig. 4). No significant differences were observed between subgroups stratified by sex, animal species, insult type and anaesthetic agent used (*p* > 0.10).

### Quality of studies

**Figure 5:**
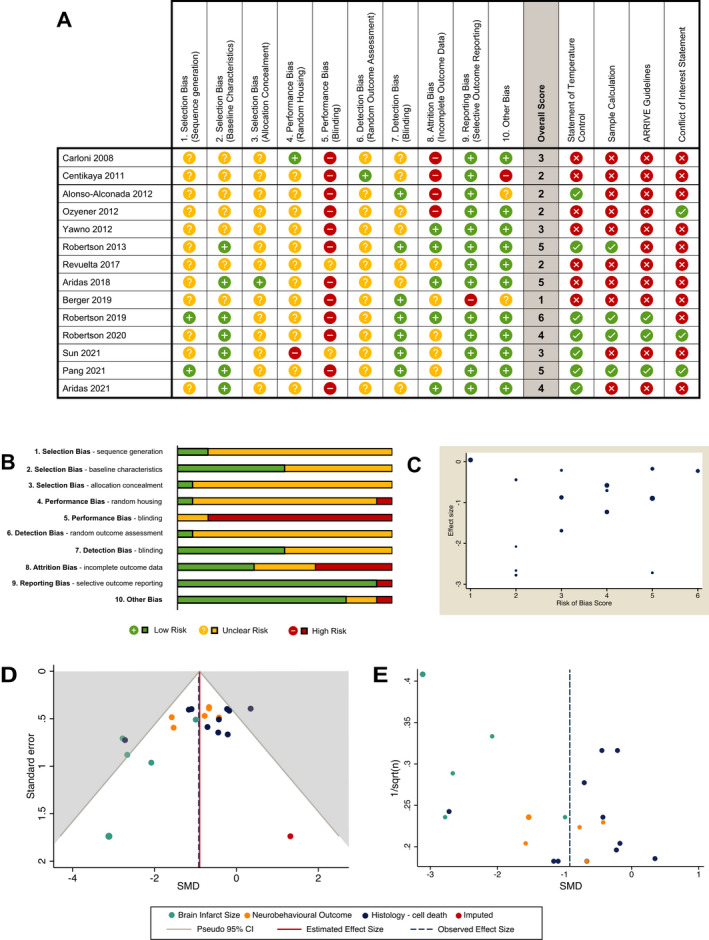
Quality of studies assessed SYRCLE RoB tool with 10 items summarised as an overall score (A and B). Influence between RoB total score and effect size shown as bubble plot (C) after meta‐regression analysis. Publication bias assess by funnel plot using SMD versus SE (D) and SMD versus 1/√*n* (E). For SMD versus SE, a trim and fill analysis was performed, with the observed pooled effect size estimate shown as a dashed line compared with the adjusted effect size (solid line) (D). SMD, standardised mean difference; SE, standard error.

The results from the risk of bias assessment are shown in Figure 5A and B. No studies were judged as low risk across all 10 domains. The median score was three (IQR 2–4.75). Of note, few studies reported the use of random sequence generation for group assignment (*n* = 2, 14.3%), allocation concealment (*n* = 1, 7.1%) or random selection for outcome assessment (*n* = 1, 7.1%). No studies reported blinding to intervention group and 12 studies (85.7%) at high risk of performance bias. Meta‐regression analysis showed no correlation between risk of bias score and effect size (*r* = 0.05, 95% CI [−0.28 to 0.37], *p* = 0.78, *I* ^2^ = 0.72) (Fig. 5C). Three studies provided a statement of adherence to ARRIVE guidelines. Seven of 14 studies reported a statement of temperature control but no significant differences in effect size was observed (*p* = 0.40) (Fig. 4). For the meta‐analysis, histology data were missing for four studies leading to high risk of attrition bias. In three studies, authors did not respond to data request and in one study^28^ only qualitative data from micrographs were available. Subgroup analysis of studies with missing data (SMD −1.95, 95% [CI −3.09 to −0.82], *p* = 0.03, *I* ^2^ = 65%) was associated with large effect size estimate compared to studies with no missing data (SMD −0.73, 95% CI [−1.13 to −0.34], *p* = 0.005, *I* ^2^ = 60%) (Fig. 4). The meta‐regression analysis also showed significant differences between studies with and without missing data (*p* = 0.03). The presence of publication bias was detected by an observed asymmetry in the funnel plot using the standard error (Fig. 5D) and a sample size‐based (1/√*n*) approach (Fig. 5E). A trim and fill analysis were performed with one imputed study resulting in an adjusted overall effect size of SMD −0.90, 95% CI (−1.23 to −0.57) from the observed (SMD −0.92, 95% CI [−1.26 to −0.58]).

### Sample size

Four studies reported sample size calculation. These were in piglet studies powered to magnetic resonance spectroscopy lactate/N‐acetyl Aspartate peak area ratio (Lac/NAA)^38^, ^39^, ^40^, ^42^ (Fig. 5).

To inform future rodent neuroprotection studies, a post hoc sample size calculation was performed for the cerebral infarct size outcome measure. The sample size required to obtain 80% power at a significance level of 0.05 for the median (2.37), 25th (1.26) and 75th centile (2.75) SMD in cerebral infarct size using the median pooled SD of 4.27 was 104 (80–362) animals.

## Discussion

This preclinical meta‐analysis demonstrates evidence of significant neuroprotection of melatonin in preclinical, term newborn animal models of perinatal HI. A key strength of the study is the strict inclusion criteria of term and near‐term animal models only. Combining 14 preclinical studies, we observed significant differences in the pooled effect size estimate, favouring melatonin in all three primary outcomes of gross cerebral infarct size, neurobehavioural outcomes and histological measure of cell death. Combined, melatonin was associated with a SMD reduction in brain injury of 0.92 (95% CI −1.26 to −0.58), which remained similar after adjusting for publication bias (adjusted SMD −0.90, 95% CI −1.23 to −0.57). This contrasts with the estimated efficacy of 0.428 (SMD) for melatonin in preclinical adult stroke models reported by McLeod et al (2005).^43^ This data are supported by several preclinical studies, which report the antioxidant^11^ and anti‐inflammatory^16^ properties of melatonin.

Importantly, the neuroprotective efficacy of melatonin remained robust in stratified subgroup analysis as a single agent and as an adjunct therapy to HT. This suggests that melatonin has the potential to be therapeutic in high and low resource settings, with and without HT. RCTs to assess novel therapies in combination with HT to improve outcomes are already underway however trials assessing other neuroprotective agents are still necessary as combined therapies with several complimentary agents targeting different aspects of the neurotoxic cascade are best placed to optimise outcomes.^44^ This meta‐analysis provides robust evidence of melatonin's potential to augment HT neuroprotection and supports the need to assess melatonin in early phase clinical trials. Although HT has been rigorously assessed in high resource settings, there is concern that HT in LMICs is not protective.^8^ The observation that melatonin is protective as a single therapy in subgroup analysis of 10 studies in this review is important and indicates potential for melatonin as a neuroprotective agent in LMICs. Infection and inflammation are significant perinatal risk factors for NE in sub‐Saharan Africa^45^ and in animal models,^46^, ^47^ inflammation‐sensitisation is known to exacerbate brain injury. Further assessment in preclinical, inflammation‐sensitisation models is also needed prior to its translation to LMICs.

Currently, there are no parenteral melatonin formulations available for intravenous use. Preclinical studies have used several excipients to enhance the solubility of melatonin. Stratified subgroup analysis highlighted improved neuroprotection in animals treated with melatonin in ethanol compared to non‐ethanol containing formulations (DMSO and a non‐ethanol based excipient). Neuroprotective effects of ethanol have been reported in newborn^39^ and adult^48^ animal models with possible mechanisms including anti‐apoptotic effects,^49^ increased HIF‐1alpha,^48^ attenuation of hyperglycolysis^50^ and free radical scavenging.^51^ While the combination of melatonin with ethanol shows promise, the translation to human trials require adherence to safe blood ethanol levels stipulated by medicines regulatory authorities. Meta‐regression analysis also showed a weak correlation between time of melatonin administration and effect size, demonstrating that outcomes improved with earlier administration of melatonin following HI. The antioxidant properties of melatonin are likely to be a key factor. Melatonin and its metabolites remove toxic reactive oxygen species formed at the time of HI; this important early effect of melatonin was elegantly shown in real‐time in newborn lambs where the cerebral efflux of free radicals was ameliorated following umbilical cord occlusion when treated with melatonin.^13^ While early melatonin administration is preferred for optimal protection, no studies administered melatonin after 2 h, therefore further studies are needed to delineate the window of opportunity. Similarly, we were unable to assess the correlation between serum melatonin levels and effect size as too few studies carried out pharmacokinetic studies. While we observed protection across all HED of melatonin, simple allometric scaling is not without its pitfalls^52^; variation in drug metabolism and genetic polymorphisms (such as CYP450 isoenzymes) between species are not considered, and varying maturity in renal and hepatic physiology in newborn models are likely to affect the accuracy of allometric scaling. Therefore, while we observed no dose effect based on HED, we are not certain whether the limitations of allometric scale may have confounded these findings. In vitro,^15^ a dose‐dependent reduction in cell death in organotypic hippocampal slices with ~0.23–22.8 mg/L of melatonin was observed, which concurred with our piglet data; serum melatonin levels of 15–30 mg/L within the first hours after HI was necessary for optimal protection.^37^, ^38^, ^39^, ^40^ Translating to clinical trials, the pharmacokinetic profile for intravenous melatonin in term newborns receiving HT requires assessment in dose‐escalating phase I studies. PK studies are only reported in preterm infants^53^, ^54^ and with enteral administration.^53^, ^55^ Extrapolating from the piglet to term infants, where body weight and the half‐life of melatonin are similar (~20 h^38^, ^55^), intravenous doses of 20 mg/kg/24 h are likely to be required.

Although this meta‐analysis reports the neuroprotective efficacy of melatonin, some caution is needed given some concerns around the quality of the studies. No studies reported low risk of bias in all domains. Few studies report evidence of sequence generation, allocation concealment and only half reported baseline characteristics. These domains have been identified as study characteristics that may exaggerate the treatment effect in clinical trials.^56^ No studies reported blinding of intervention during the experiment, which may introduce bias to the neurocritical care management of animals and therefore neurological outcomes. While blinding may not be feasible in all interventions, researchers should, where possible, design animal studies to minimise these confounding factors. It is important to note that the study quality scores reflect inadequate reporting as clear evidence in the publication was required for a domain to be classified as low risk of bias. Despite the publication of the ARRIVE guidelines in 2010 to improve reporting of preclinical studies, statement of adherence was only observed in three publications since its inception. Of note, only four studies (limited to the piglet model) reported sample calculations to ensure studies were adequately powered. These studies used Lac/NAA to power the study. The use of sample size calculations in rodent studies is uncommon, therefore we performed a sample size estimation on the most frequently used outcome marker, cerebral infarct size to inform future study design. This showed that over 100 rodents are needed for the median SMD. We also attempted to estimate the sample size required for neurobehavioural outcomes however this was not feasible as the studies used different neurobehavioural assessment methods.

There are limitations to this meta‐analysis. First, the histology data were aggregated into a single nested outcome to allow for subgroup analysis. While this is a conservative approach to avoid inflation of the effect size estimate, we acknowledge that regional neuroprotective effects may be concealed. In the piglet model, we observed no difference in TUNEL‐positive cell counts overall, however there was significant regional protection in the sensorimotor cortex, a vulnerable region of high metabolism.^37^, ^40^ We also acknowledge that histological data were missing for three studies where authors did not respond to data request. This was associated with a larger SMD in subgroup analysis therefore we cannot exclude that data attrition may contribute to an overestimation of the effect size. However, the effect size remained significant, albeit smaller in studies with no missing data. In the presence of small sample size, the use of SMD may introduce measurement error, but this was unavoidable given the heterogeneous nature of preclinical studies. The assessment of publication bias when using SMD is also complex, with reports of overestimation in publication bias in when the sample size is small using SMD versus SE funnel plots.^27^ We used a sample size‐based precision estimate (1/√*n*) approach as recommended by Zwetsloot et al,^27^ to confirm the presence of asymmetry and publication bias, which highlights the need to interpret the overall pooled SMD with caution. Our meta‐analysis was also limited to histopathological and animal neurobehavioural outcomes, which are inherently heterogeneous in nature and therefore difficult to combine. Further sources of heterogeneity include variability across study design, drug administration protocols and the effect of animal species. We overcame this by using a random effects model, which estimates the mean distribution of effects. We also observed no significant influences of sex, species, insult type and anaesthetic agents although some subgroups were moderately heterogeneous so should be cautiously interpreted. For example, we cannot exclude the influence of species on the larger effect size in cerebral infarct (exclusive rodents) compared to cell death (mixed large and small animals). Nevertheless, despite these limitations, the study still highlighted a positive pooled outcome in all subgroup analyses.

We suggest the need for a more collaborative approach to standardise outcome measurement for preclinical neuroprotection studies. Translation biomarkers such as proton magnetic resonance spectroscopy Lac/NAA peak area ratio and amplitude‐integrated electroencephalography have been increasingly used in animal neuroprotection studies,^29^, ^37^, ^38^, ^39^, ^40^ validated in the clinical setting^57^ to expedite early assessment of the biological effect. We have not included this surrogate biomarker in the meta‐analysis to avoid publication bias as these were largely limited to studies from our group (*n* = 4) and one other study.^29^ Nonetheless, Lac/NAA holds great promise as a standard primary outcome measure for preclinical neuroprotection studies and may be used to estimate the sample size. A standardised approach would reduce heterogeneity, improve the quality of future preclinical meta‐analyses, and improve the translational relevance of animal studies.

In conclusion, this meta‐analysis provides strong preclinical evidence of the neuroprotective efficacy in melatonin as a postnatal treatment for HI in term newborn animals, supporting the translation into clinical trials for babies with NE. Melatonin was effective at augmenting HT neuroprotection and as a single therapy after HI, making it relevant to high‐ and low‐income settings respectively. The neuroprotective benefit of melatonin appears to be time critical, and the use of ethanol excipients augments neuroprotection, however we did not observe a dose response based on simple allometric scaling. Early treatment with melatonin provided better protection, which resembles the findings with HT. Early cooling <3 h after birth provided better protection compared to delayed treatment at 3–6 h.^58^ It is likely that melatonin needs to be initiated as early as possible in clinical trials before the onset of secondary energy failure. The ease of administration of intravenous melatonin will enable out‐born babies to receive treatment as soon as possible after birth while awaiting transfer to specialist cooling centres for HT. Preclinical studies defining the therapeutic window for melatonin therapy are needed and the potential benefit of melatonin in inflammation sensitised models is unclear, although likely.^59^ Finally, these data demonstrate that closer collaboration between translational researchers is needed to standardise outcomes to reduce heterogeneity between studies, improve the quality of preclinical meta‐analysis and links with clinical researchers are important to ensure the design of clinical trials is optimal.

## Conflict of Interest

None declared.

## Author Contributions

Raymand Pang and Nicola J. Robertson conceptualised and designed the study. Raymand Pang and Hyun Jee Han carried out the literature search, data extraction and quality assessment of the studies. Christopher Meehan acted as the third reviewer to resolve discrepancies during screening. Raymand Pang carried out the statistical analysis, organised and wrote the first draft of the manuscript. Nicola J. Robertson secured funding, supervised and assisted in the interpretation and write up of the data. Suzanne L. Miller contributed to the data and together with Xavier Golay, provided valuable input to analysis of the data and the manuscript. All authors reviewed and approval the final version of the manuscript and agreed to be accountable for all aspects of the work.

## Supporting information


**Data S1.** Systematic review protocol (based on the SYRCLE template), and the full search terms used in the literature search
