# Supplementary material for: Efficacy of melatonin in term neonatal models of perinatal hypoxia‐ischaemia
Source: Ann Clin Transl Neurol. 2022 Apr 12;9(6):795–809. doi: 10.1002/acn3.51559 (PMC9186150; doi:10.1002/acn3.51559)
Supplement: Supplementary file 1 — Data S1. Systematic review protocol (based on the SYRCLE template), and the full search terms used in the literature search [file ACN3-9-795-s001.docx]

| 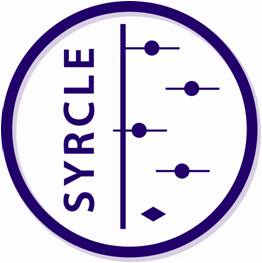 **Systematic Review Protocol for Animal Intervention Studies**  **Format by SYRCLE (**[**www.syrcle.nl**](http://www.syrcle.nl)**)**  **Version 2.0 (December 2014)** | | | | |
| --- | --- | --- | --- | --- |
| **Item #** | **Section/Subsection/Item** | **Description** | | **Check for approval** |
|  | A. General | | | |
| 1. | Title of the review | Preclinical systematic review of melatonin for neonatal encephalopathy | | NJR |
| 2. | Authors (names, affiliations, contributions) | R Pang^1^, H Han^1^, C Meehan^1^, X Golay^1^, S Miller^2^, NJ Robertson^1,3^  ^1^ University College London  ^2^ University of Monash  ^3^ University of Edinburgh | | NJR |
| 3. | Other contributors (names, affiliations, contributions) |  | | NJR |
| 4. | Contact person + e-mail address | R Pang, [r.pang@ucl.ac.uk](mailto:r.pang@ucl.ac.uk) | | NJR |
| 5. | Funding sources/sponsors | Gates Foundation  Wellbeing of Women | | NJR |
| 6. | Conflicts of interest | None declared | | NJR |
| 7. | Date and location of protocol registration | 30/3/2021, R Pang, UCL | | NJR |
| 8. | Registration number (if applicable) | N/A | | NJR |
| 9. | Stage of review at time of registration | N/A | | NJR |
|  | B. Objectives | | | |
|  | Background | | | |
| 10. | What is already known about this disease/model/intervention? Why is it important to do this review? | Not all babies benefit from therapeutic hypothermia (HT), the only treatment currently available in the high resource setting for moderate to severe NE. 30-50% infants still develop adverse neurodevelopmental outcomes and the rates of cerebral palsy remains static at 19%. Adjunct therapies to HT are urgently needed in the high income setting to improve outcomes, requiring well powered RCTs. In the low resource setting, the benefit of HT remains unclear (Thayyil et al., 2021) and alternative neuroprotective interventions are needed.  Melatonin has shown promise is several preclinical studies as a safe and effective neuroprotective therapy in combination with HT (Robertson 2013, 2019, 2020, Pang 2021, Aridas 2018), however this has not yet translated to the bedside for babies with NE. A meta-analysis of clinical studies show studies are limited to underpowered pilot data, using enteral route of administration with few reporting relevant neurodevelopmental outcomes. Intravenous routes are likely needed to harness the full neuroprotective benefit of melatonin and clinical trials are urgently needed. This preclinical meta-analysis will provide further evidence to support the need to take melatonin through the translational pipeline, aiming to assess the efficacy in term animal models of NE. | | NJR |
|  | Research question | | | |
| 11. | Specify the disease/health problem of interest | Neonatal Encephalopathy AND/OR Hypoxic Ischaemic Encephalopathy (component of hypoxia and ischaemia) | | NJR |
| 12. | Specify the population/species studied | Term newborn babies, animal model equivalent | | NJR |
| 13. | Specify the intervention/exposure | Melatonin as primary neuroprotective agent | | NJR |
| 14. | Specify the control population | Normothermic (NT) or hypothermic (HT) animals not receiving melatonin | | NJR |
| 15. | Specify the outcome measures | - Outcome 1: infarct size (global), - Outcome 2: neurobehavioural outcomes, or - Outcome 3: immunohistochemistry (regional) - marker of cell death | | NJR |
| 16. | State your research question (based on items 11-15) | Overall: Does melatonin improve neurological outcomes compared to control animals (HT or NT) in term equivalent animal models of NE?  Does melatonin improve outcomes further in combination with HT compared to HT alone in NE? (relevant to the high resource setting)  Does melatonin improve outcomes as a monotherapy compared to NT animals in NE? | | NJR |
|  | C. Methods | | | |
|  | Search and study identification | | | |
| 17. | Identify literature databases to search (*e.g.* Pubmed, Embase, Web of science) | MEDLINE via PubMed Web of Science  □SCOPUS EMBASE  □Other, namely:  □Specific journal(s), namely: | | NJR |
| 18. | Define electronic search strategies (*e.g.* use the [step by step search guide^15^](http://www.ncbi.nlm.nih.gov/pmc/articles/PMC3265183/pdf/LA-11-087.pdf) and animal search filters[^20,^](http://www.ncbi.nlm.nih.gov/pmc/articles/PMC3104815/pdf/LA-09-117.pdf) [^21^](http://lan.sagepub.com/content/48/1/88.full.pdf+html)) | When available, please add a supplementary file containing your search strategy: **Literature Search Strategies.docx** | | NJR |
| 19. | Identify other sources for study identification | □Reference lists of included studies □Books  □Reference lists of relevant reviews  □Conference proceedings, namely:  □Contacting authors/ organisations, namely:  □Other, namely: | | NJR |
| 20. | Define search strategy for these other sources | N/A | | NJR |
|  | Study selection | | | |
| 21. | Define screening phases (*e.g.* pre-screening based on title/abstract, full text screening, both) | 1. Stage 1: Pre-screening of title using SyrF platform 2. Stage 2: Full text screening | | NJR |
| 22. | Specify (a) the number of reviewers per screening phase and (b) how discrepancies will be resolved | 2 reviewers – RP and HH  Third reviewer – CM to resolve discrepancies | | NJR |
|  | *Define all inclusion and exclusion criteria based on:* | | | |
| 23. | Type of study (design) | Inclusion criteria: preclinical, in-vivo studies  Exclusion criteria: clinical studies, ex-vivo or in-vitro studies | | NJR |
| 24. | Type of animals/population (*e.g.* age, gender, disease model) | Inclusion criteria: Term equivalent age animal models (newborn piglet, sheep > 126 gestation, rodent models (at least P7 in rodents). Both Hypoxia and ischaemic component  Exclusion criteria: Preterm models and adult models, ischaemia only models (stroke models), in-vitro and ex-vivo studies | | NJR |
| 25. | Type of intervention (*e.g.* dosage, timing, frequency) | Inclusion criteria: Melatonin alone, or in combination with HT  Exclusion criteria: melatonin not used | | NJR |
| 26. | Outcome measures | Inclusion criteria: (i) overall infarct size, (ii) neurobehavioural outcome, and (iii) histological assessment of cell death  Exclusion criteria: histology not assessing cell death | | NJR |
| 27. | Language restrictions | Inclusion criteria: English  Exclusion criteria: English | | NJR |
| 28. | Publication date restrictions | Inclusion criteria: No restriction  Exclusion criteria: - | | NJR |
| 29. | Other | Inclusion criteria: -  Exclusion criteria: - | | NJR |
| 30. | Sort and prioritize your exclusion criteria per selection phase | Selection phase: Screening of abstract  1. Melatonin as treatment arm  2. Preclinical studies only, exclude human studies  3. In vivo studies only, exclude in vitro or ex vivo  4. Animal model of HI (NE), exclude stroke models  5. Term equivalent model, exclude preterm models  6. One of the three outcome measures present, excluded if non are present  Selection phase: Full text screening  1. Exclude preterm models, adult models  2. Exclude stroke models, need both HI components  3. At least one of the three outcome measure present | | NJR |
|  | Study characteristics to be extracted (for assessment of external validity, reporting quality) | | | |
| 31. | Study ID (*e.g.* authors, year) | Author, Year, Title, Journal Citation | | NJR |
| 32. | Study design characteristics (*e.g.* experimental groups, number of animals) | Number of experiment groups, number of animals per group, temperature control (HT or NT, type of HT device used), anaesthetic agent used, duration of experiment | | NJR |
| 33. | Animal model characteristics (*e.g.* species, gender, disease induction) | Age / gestation  Species  Mean weight  Sex – male / female / mixed / not stated  HI (method, duration, FiO2) | | NJR |
| 34. | Intervention characteristics (*e.g.* intervention, timing, duration) | Time of first dose  Dose in mg/kg per dose, and in first 24h. Given different species anticipated, to be converted to human equivalent dose  Number of doses per animal, number of doses per day  Route  Excipient and %  PK study – blood melatonin levels (Cmax) | | NJR |
| 35. | Outcome measures | Outcome 1: infarct size as percentage, proportion or raw values (mean, error, SD/SE, n), method of measuring infarct size  Outcome 2: neurobehavioural outcomes  Type of test performed, timing of test performed, quantitative value (mean, error, SD/SE, n)  Outcome 3: immunohistochemistry - marker of cell death  Type of marker to quantify cell death (eg TUNEL, CC3, NeuN), region assessed (mean, error, SD/SE, n) | | NJR |
| 36. | Other (*e.g.* drop-outs) |  | |  |
|  | Assessment risk of bias (internal validity) or study quality | | | |
| 37. | Specify (a) the number of reviewers assessing the risk of bias/study quality in each study and (b) how discrepancies will be resolved | 2 reviewers – RP and HH  Third reviewer – CM to resolve discrepancies | | NJR |
| 38. | Define criteria to assess (a) the internal validity of included studies (*e.g.* selection, performance, detection and attrition bias) and/or (b) other study quality measures (*e.g.* reporting quality, power) | □By use of [SYRCLE's Risk of Bias tool^4^](http://www.biomedcentral.com/1471-2288/14/43/abstract)  By use of SYRCLE’s Risk of Bias tool, adapted as follows: addition of - Statement of arrive guidance followed, statement of temperature control, sample size, conflict of interest statement  □By use of [CAMARADES' study quality checklist, e.g ^22^](http://www.ncbi.nlm.nih.gov/pubmed/15060322)  □By use of CAMARADES' study quality checklist, adapted as follows:  □Other criteria, namely: | | NJR |
|  | Collection of outcome data | | | |
| 39. | For each outcome measure, define the type of data to be extracted (*e.g.* continuous/dichotomous, unit of measurement) | Outcome 1: Continuous - infarct size as percentage, proportion or raw values (mean, error, SD/SE, n), method of measuring infarct size  Outcome 2: Continuous - neurobehavioural outcomes  Type of test performed, timing of test performed, quantitative value depending on test (mean, error, SD/SE, n)  Outcome 3: Continuous - immunohistochemistry - marker of cell death  Type of marker to quantify cell death (eg TUNEL, CC3, NeuN), region assessed (mean, error, SD/SE, n) | | NJR |
| 40. | Methods for data extraction/retrieval (*e.g.* first extraction from graphs using a digital screen ruler, then contacting authors) | Raw values extracted from papers / tables to maintain precision of data available  Authors contacted for raw values if graphical data present only | | NJR |
| 41. | Specify (a) the number of reviewers extracting data and (b) how discrepancies will be resolved | 2 reviewers – RP and HH  Review of data extracted by both reviewers and if discrepencies, to review full text and discuss until consensus reached | | NJR |
|  | Data analysis/synthesis | | | |
| 42. | Specify (per outcome measure) how you are planning to combine/compare the data (*e.g.* descriptive summary, meta-analysis) | For all three outcome measures: (i) overall infarct size, (ii) neurobehavioural outcome, and (iii) histological assessment of cell death, standardised mean difference (SMD) will be used due to differences in units between studies  Combination of outcomes to form nested outcomes as described by Vesterinen et al (2014) and used in the stroke melatonin meta-analysis by McLeod et al (2005)  Random effects model (DerSimmonian and Laird) to pool the data by (a) outcome measure (b) combined for all outcome measures  Specifically:  Outcome 1: SMD and Random effects  Outcome 2: For each test, the final time point of measurement will be used. If several tests performed, the outcomes will be combined to form a nested outcome for each study  Outcome 3: Regional counts collected and combined to form a nested outcomes for each study. Overall counts (if present) will be excluded to avoid double counting  When performing meta-analysis we will pool data by outcome measure initially  For subgroup analysis, a nested outcomes for each study will be deduced which includes all available data from the study (eg combining outcome 1 and outcome 2 and outcome 3 as available) | | NJR |
| 43. | Specify (per outcome measure) how it will be decided whether a meta-analysis will be performed | Meta-analysis will be performed for each outcome measure. Heterogeneity will be assessed. | | NJR |
|  | *If a meta-analysis seems feasible/sensible, specify (for each outcome measure):* | | | |
| 44. | The effect measure to be used (*e.g.* mean difference, standardized mean difference, risk ratio, odds ratio) | Standardised mean difference | | NJR |
| 45. | The statistical model of analysis (*e.g.* random or fixed effects model) | Random effects model - DerSimmonian and Laird | | NJR |
| 46. | The statistical methods to assess heterogeneity (*e.g.* I^2^, Q) | I^2^ | | NJR |
| 47. | Which study characteristics will be examined as potential source of heterogeneity (subgroup analysis) | 1. Melatonin over 24h, converted to human equivalent dose  2. Time to first dose  3. Small vs Large animal studies  4. Type of insult  5. Sex  6. Anaesthetic use  7. Excipient in melatonin formulation  8. HT vs NT studies  9. RoB score  10. Statement of temperature control | | NJR |
| 48. | Any sensitivity analyses you propose to perform | N/A | | NJR |
| 49. | Other details meta-analysis (*e.g.* correction for multiple testing, correction for multiple use of control group) | N/A | | NJR |
| 50. | The method for assessment of publication bias | Funnel plot with trim and fill analysis | | NJR |
|  | | | | |
| Final approval by (names, affiliations):  NJ Robertson, UCL | |  | Date: 30/3/21 | |

**Literature Search Strategy**

**Database: Embase**

1. Melatonin.mp. or exp *melatonin/
2. exp brain injury/ or neonatal encephalopathy.mp.
3. exp hypoxia/ or exp hypoxia ischemia/ or hypoxia-isch*mia.mp. or exp brain damage/
4. exp hypoxia/ or hypoxia.mp. or exp brain hypoxia/ or exp fetus hypoxia/ or exp experimental hypoxia/ or exp newborn hypoxia/
5. exp perinatal asphyxia/ or asphyxia.mp. or exp asphyxia/
6. exp animal experiment/ or exp animal model/ or exp experimental animal/ or exp transgenic animal/ or exp male animal/ or exp female animal/ or exp juvenile animal/ or animal/ or chordata/ or vertebrate/ or tetrapod/ or exp fish/ or amniote/ or exp amphibia/ or mammal/ or exp reptile/ or exp sauropsid/ or therian/ or exp monotremate/ or placental mammals/ or exp marsupial/ or Euarchontoglires/ or exp Afrotheria/ or exp Boreoeutheria/ or exp Laurasiatheria/ or exp Xenarthra/ or primate/ or exp Dermoptera/ or exp Glires/ or exp Scandentia/ or Haplorhini/ or exp prosimian/ or simian/ or exp tarsiiform/ or Catarrhini/ or exp Platyrrhini/ or ape/ or exp Cercopithecidae/ or hominid/ or exp hylobatidae/ or exp chimpanzee/ or exp gorilla/ or exp orang utan/ or (animal or animals or pisces or fish or fishes or catfish or catfishes or sheatfish or silurus or arius or heteropneustes or clarias or gariepinus or fathead minnow or fathead minnows or pimephales or promelas or cichlidae or trout or trouts or char or chars or salvelinus or salmo or oncorhynchus or guppy or guppies or millionfish or poecilia or goldfish or goldfishes or carassius or auratus or mullet or mullets or mugil or curema or shark or sharks or cod or cods or gadus or morhua or carp or carps or cyprinus or carpio or killifish or eel or eels or anguilla or zander or sander or lucioperca or stizostedion or turbot or turbots or psetta or flatfish or flatfishes or plaice or pleuronectes or platessa or tilapia or tilapias or oreochromis or sarotherodon or common sole or dover sole or solea or zebrafish or zebrafishes or danio or rerio or seabass or dicentrarchus or labrax or morone or lamprey or lampreys or petromyzon or pumpkinseed or pumpkinseeds or lepomis or gibbosus or herring or clupea or harengus or amphibia or amphibian or amphibians or anura or salientia or frog or frogs or rana or toad or toads or bufo or xenopus or laevis or bombina or epidalea or calamita or salamander or salamanders or newt or newts or triturus or reptilia or reptile or reptiles or bearded dragon or pogona or vitticeps or iguana or iguanas or lizard or lizards or anguis fragilis or turtle or turtles or snakes or snake or aves or bird or birds or quail or quails or coturnix or bobwhite or colinus or virginianus or poultry or poultries or fowl or fowls or chicken or chickens or gallus or zebra finch or taeniopygia or guttata or canary or canaries or serinus or canaria or parakeet or parakeets or grasskeet or parrot or parrots or psittacine or psittacines or shelduck or tadorna or goose or geese or branta or leucopsis or woodlark or lullula or flycatcher or ficedula or hypoleuca or dove or doves or geopelia or cuneata or duck or ducks or greylag or graylag or anser or harrier or circus pygargus or red knot or great knot or calidris or canutus or godwit or limosa or lapponica or meleagris or gallopavo or jackdaw or corvus or monedula or ruff or philomachus or pugnax or lapwing or peewit or plover or vanellus or swan or cygnus or columbianus or bewickii or gull or chroicocephalus or ridibundus or albifrons or great tit or parus or aythya or fuligula or streptopelia or risoria or spoonbill or platalea or leucorodia or blackbird or turdus or merula or blue tit or cyanistes or pigeon or pigeons or columba or pintail or anas or starling or sturnus or owl or athene noctua or pochard or ferina or cockatiel or nymphicus or hollandicus or skylark or alauda or tern or sterna or teal or crecca or oystercatcher or haematopus or ostralegus or shrew or shrews or sorex or araneus or crocidura or russula or european mole or talpa or chiroptera or bat or bats or eptesicus or serotinus or myotis or dasycneme or daubentonii or pipistrelle or pipistrellus or cat or cats or felis or catus or feline or dog or dogs or canis or canine or canines or otter or otters or lutra or badger or badgers or meles or fitchew or fitch or foumart or foulmart or ferrets or ferret or polecat or polecats or mustela or putorius or weasel or weasels or fox or foxes or vulpes or common seal or phoca or vitulina or grey seal or halichoerus or horse or horses or equus or equine or equidae or donkey or donkeys or mule or mules or pig or pigs or swine or swines or hog or hogs or boar or boars or porcine or piglet or piglets or sus or scrofa or llama or llamas or lama or glama or deer or deers or cervus or elaphus or cow or cows or bos taurus or bos indicus or bovine or bull or bulls or cattle or bison or bisons or sheep or sheeps or ovis aries or ovine or lamb or lambs or mouflon or mouflons or goat or goats or capra or caprine or chamois or rupicapra or leporidae or lagomorpha or lagomorph or rabbit or rabbits or oryctolagus or cuniculus or laprine or hares or lepus or rodentia or rodent or rodents or murinae or mouse or mice or mus or musculus or murine or woodmouse or apodemus or rat or rats or rattus or norvegicus or guinea pig or guinea pigs or cavia or porcellus or hamster or hamsters or mesocricetus or cricetulus or cricetus or gerbil or gerbils or jird or jirds or meriones or unguiculatus or jerboa or jerboas or jaculus or chinchilla or chinchillas or beaver or beavers or castor fiber or castor canadensis or sciuridae or squirrel or squirrels or sciurus or chipmunk or chipmunks or marmot or marmots or marmota or suslik or susliks or spermophilus or cynomys or cottonrat or cottonrats or sigmodon or vole or voles or microtus or myodes or glareolus or primate or primates or prosimian or prosimians or lemur or lemurs or lemuridae or loris or bush baby or bush babies or bushbaby or bushbabies or galago or galagos or anthropoidea or anthropoids or simian or simians or monkey or monkeys or marmoset or marmosets or callithrix or cebuella or tamarin or tamarins or saguinus or leontopithecus or squirrel monkey or squirrel monkeys or saimiri or night monkey or night monkeys or owl monkey or owl monkeys or douroucoulis or aotus or spider monkey or spider monkeys or ateles or baboon or baboons or papio or rhesus monkey or macaque or macaca or mulatta or cynomolgus or fascicularis or green monkey or green monkeys or chlorocebus or vervet or vervets or pygerythrus or hominoidea or ape or apes or hylobatidae or gibbon or gibbons or siamang or siamangs or nomascus or symphalangus or hominidae or orangutan or orangutans or pongo or chimpanzee or chimpanzees or pan troglodytes or bonobo or bonobos or pan paniscus or gorilla or gorillas or troglodytes).ti,ab.
7. **2 OR 3 OR 4 OR 5**
8. **1 AND 6 AND 7**

**Database: MEDLINE**

1. melatonin.mp. or exp *Melatonin/
2. exp Hypoxia-Ischemia, Brain/ or neonatal encephalopathy.mp. or exp Cerebral Palsy/ or exp Asphyxia Neonatorum/
3. exp Fetal Hypoxia/ or exp Hypoxia, Brain/ or exp Hypoxia-Ischemia, Brain/ or hypoxia.mp. or exp Hypoxia/
4. exp Hypoxia-Ischemia, Brain/ or hypoxia-ischemia.mp. or exp Hypoxia, Brain/
5. exp Asphyxia Neonatorum/ or exp Asphyxia/ or asphyxia.mp.
6. 2 or 3 or 4 or 5
7. 1 and 6

**Database: Web of Science**

1. Melatonin
2. **TOPIC:** (neonatal encephalopathy) *OR* **TOPIC:** (Hypoxia-ischaemia) *OR* **TOPIC:** (Hypoxia) *OR* **TOPIC:** (cerebral palsy) *OR* **TOPIC:** (asphyxia) *OR* **TOPIC:** (asphyxia neonatorum) *OR* **TOPIC:** (fetal hypoxia) *OR* **TOPIC:** (Brain hypoxia) *OR* **TOPIC:** (brain hypoxia-ischemia)
3. 1 and 2
